# Effect of photobiomodulation therapy on pain perception during anesthetic puncture of dental local anesthesia: A systematic review

**DOI:** 10.1016/j.clinsp.2023.100322

**Published:** 2024-03-13

**Authors:** Caio Melo Mesquita, Millena Barroso Oliveira, Marcelo Dias Moreira de Assis Costa, Walbert Andrade Vieira, Rafael Rodrigues Lima, Sigmar de Mello Rode, Luiz Renato Paranhos

**Affiliations:** a School of Dentistry, Universidade Federal de Uberlândia (UFU), Uberlândia, MG, Brazil; b Postgraduate Program in Dentistry, School of Dentistry, Universidade Federal de Uberlândia (UFU), Uberlândia, MG, Brazil; c Department of Restorative Dentistry, Endodontics Division, School of Dentistry, Universidade Estadual de Campinas (UNICAMP), Piracicaba, SP, Brazil; d Laboratory of Functional and Structural Biology, Institute of Biological Sciences, Universidade Federal do Pará (UFPA), Belém, PA, Brazil; e Department of Dental Materials and Prosthetics, Institute of Science and Technology, Universidade Estadual Paulista Júlio de Mesquita Filho (UNESP), São Paulo, SP, Brazil; f Division of Social and Preventive Dentistry, School of Dentistry, Universidade Federal de Uberlândia (UFU), Uberlândia, MG, Brazil

**Keywords:** Dental anesthesia, Local anesthesia, Low-level light therapy, Pain perception, Photobiomodulation therapy

## Abstract

**Background:**

Local anesthetic puncture is often related to the experience of pain. This study aimed to systematically analyze the literature on changes in pain perception during the anesthetic puncture of dental local anesthesia after Photobiomodulation Therapy (PBMT).

**Material and methods:**

An electronic search was performed in eight primary databases (Embase, LILACS, BBO, LIVIVO, MedLine *via* PubMed, SciELO, Scopus, and Web of Science) and three additional ones (EASY, Google Scholar, and OATD) to partially capture the “gray literature”. The PICO strategy was used to identify randomized clinical trials evaluating the analgesic effect of PBMT in the anesthetic puncture site of dental local anesthesia compared to placebo or control groups, without restrictions on publication language and year. Two reviewers extracted the data and assessed the individual risk of bias of the eligible studies using the Cochrane Collaboration Risk of Bias Tool version 2.0.

**Results:**

The electronic search found 3,485 records, of which eight met the eligibility criteria and were included in the qualitative synthesis. The studies were published from 2011 to 2022. None of the included studies had a low risk of bias. PBMT groups showed no significant difference in pain scores compared to placebo and control groups of most studies.

**Conclusion:**

Based on a low to very low certainty of evidence, PBMT seems to have no effect on pain perception during anesthetic puncture in patients undergoing dental local anesthesia.

## Highlights

• Photobiomodulation therapy seems to have little or no effect on pain perception during the anesthetic puncture in patients undergoing dental local anesthesia.
• Clinical trials found in the literature used different study samples, pain assessment tools, and photobiomodulation therapy protocols.
• Further randomized studies should be performed with a standardized methodology to strengthen the current evidence.

## Introduction

Pain is defined as “an unpleasant sensory and emotional experience associated with actual or potential tissue damage or described in terms of such damage”. Furthermore, it considers a sum of the present and past experiences of an individual [1]. Most dental procedures require local anesthesia, including extractions, pulpotomies, root canal treatments/pulpectomies, abscess drainage, and oral surgeries [2]. Local anesthetics are nevertheless associated with pain, which may be exacerbated by the fear and anxiety caused by the anesthetic puncture [2]. Several factors that can influence pain perception may be hard to assess. Hence, different methods play this role [2], [3], [4], [5], [6], [7]: objective measurement tools, such as Visual Analog Scale (VAS) [7], Visual Numerical Scale (VNS) [6], Wong-Baker Faces Pain Rating Scale (WBFPRS) [5], and Numerical Rating Scale (NRS) [4]; and subjective assessment tools, such as Face, Legs, Activity, Cry, and Consolability (FLACC) scale [3], and Sound, Eyes, and Motor (SEM) scale [2].

Generally, there are resources that can be used to modulate pain perception, such as photobiomodulation therapy (PBMT) [8]. PBMT is a technology performed mainly with Light-Emitting Diodes (LEDs) and is based on the non-ionizing radiation of the photobiomodulation mechanism of red light and near-infrared [8]. There are suggested pathways for this mechanism, and the primary hypothesis is that body tissues absorb light and reach cytochrome C oxidase (chromophores present in mitochondria), inducing increased ATP production and accelerating cellular metabolism [8]. Depending on the PBMT device settings and application protocols, this molecular process may trigger beneficial clinical effects, such as inflammatory process modulation, improved tissue repair and regeneration, and analgesia [8].

In Dentistry, PBMT is applied to a wide range of procedures to make them more practical and comfortable [9], [10], [11], [12], [13], [14], [15], [16], [17], [18], [19]. On the one hand, the literature has not fully supported some expected effects of PBMT, such as chronic pain management in temporomandibular disorder patients [10], long-term relief of xerostomia and hyposalivation [12], stability of orthodontic mini-implants [14], orthodontic movement acceleration [15], and denture stomatitis treatment compared to conventional interventions [17]. On the other hand, PBMT is strongly supported regarding the management of acute symptoms, such as pain related to fixed orthodontic appliances [9], postoperative pain from third molar extraction [14], pain and trismus from orthognathic surgery [13], and pain-related symptoms after the first weeks of tooth whitening [16].

Although there are primary studies about the analgesic effect of photobiomodulation therapy on dental local anesthetic puncture, consolidating the scientific evidence is still necessary to provide a rational basis for clinical decisions. Therefore, this review aimed to systematically analyze the literature on changes in pain perception during the anesthetic puncture of dental local anesthesia after PBMT.

## Material and methods

### Protocol registration

The protocol was reported according to the Preferred Reporting Items for Systematic Review and Meta-Analysis Protocols (PRISMA-P) [18] and registered in the International Prospective Register of Systematic Reviews (PROSPERO) database under number CRD42022304740 (https://www.crd.york.ac.uk/PROSPERO/). This systematic review was reported following the Preferred Reporting Items for Systematic Reviews and Meta-Analyses (PRISMA) [19] and conducted according to the Joanna Briggs Institute (JBI) Manual.

### Research question and eligibility criteria

The review was designed to answer the following question: “Is PBMT effective in reducing pain perception during anesthetic puncture in patients undergoing dental local anesthesia compared to placebo or topical anesthetic?” following the **PICO** framework: **P** (population), **I** (intervention), **C** (comparison), and **O** (outcome).

Inclusion criteria

• Population: Patients of all ages undergoing dental local anesthesia, regardless of anesthetic technique;
• Intervention: PBMT at the puncture site for analgesic effect before dental local anesthetic puncture;
• Comparator: Control groups that received a placebo, no pre-anesthetic intervention, or topical anesthetic;
• Outcome: Pain perception during dental local anesthetic puncture;
• Study design: Randomized clinical trials.

There were no restrictions on publication language or year.

Exclusion criteria

• Studies with samples of patients with chronic systemic diseases, immunocompromised, and with acute or recurrent dental infections that compromise pain perception (i.e., irreversible pulpitis and phoenix abscess);
• Studies with sample overlapping (in this case, considering the most recent study and best described the methodology and results);
• Books, book chapters, case reports, case series, congress papers, editorials, letters to the editor, literature reviews, qualitative studies, and studies with animals.

### Sources of information, search, and selection of studies

The electronic searches were performed on Noveminr 2021 in Embase, LILACS (Latin American and Caribbean Health Science Literature), BBO (Brazilian Bibliography of Odontology), LIVIVO, MedLine (via PubMed), and SciELO, and the Scopus and Web of Science citation databases. The EASY, Google Scholar, and Open Access Theses and Dissertations (OATD) databases partially captured the “gray literature”. These steps were performed to minimize the selection bias. The MedLine search was constantly updated with electronic alerts until June 2022. The search descriptors were selected according to the MeSH (Medical Subject Headings), DeCS (Health Sciences Descriptors), and Emtree (Embase Subject Headings) resources. The Boolean operators "AND" and "OR" promoted several combinations among the descriptors, respecting the syntax rules of each database. Table 1 shows more details of search strategies and databases.

**Table 1:** Strategies for database search.

Databases	Search Strategy (November 2021) and Update (June 2022)
**Main Databases**	
Embase https://www.embase.com	('lasers' OR 'laser therapy' OR 'low level laser therapy' OR 'low level light therapy' OR 'photobiomodulation' OR 'photobiomodulation therapy' OR 'laser biostimulation' OR 'laser phototherapy') AND ('pain' OR 'pain management' OR 'pain perception' OR 'pain measurement') AND ('anesthesia' OR 'dental anesthesia' OR 'surgery, oral' OR 'oral surgical procedures' OR 'oral surgery' OR 'oral procedure' OR 'dental surgery' OR 'dental procedures')
LILACS and BBO http://pesquisa.bvsalud.org/	**/pt** (("Lasers" OR "Terapia a Laser" OR "Terapia com Luz de Baixa Intensidade" OR "Bioestimulação a Laser" OR "Irradiação a Laser de Baixa Intensidade" OR "Irradiação a Laser de Baixa Potência" OR "Terapia a Laser de Baixa Intensidade" OR "Terapia a Laser de Baixa Potência") AND ("Dor" OR "Manejo da Dor" OR "Percepção da Dor" OR "Mensuração da Dor") AND (“anestesia” OR “anestesia dentária” OR "Cirurgia Bucal" OR "Procedimentos Cirúrgicos Bucais" OR "Cirurgia Oral" OR "Procedimentos Orais")) AND (db:("LILACS" OR "BBO"))
	**/en** (("Lasers" OR "Laser Therapy" OR "Low Level Laser Therapy" OR "Low Level Light Therapy" OR "Photobiomodulation" OR "Photobiomodulation Therapy" OR "Laser Biostimulation" OR "Laser Phototherapy") AND ("Pain" OR "Pain Management" OR "Pain Perception" OR "Pain Measurement") AND ("Anesthesia" OR "Anesthesia, Dental" OR "Surgery, Oral" OR "Oral Surgical Procedures" OR "Oral Surgery" OR "Oral Procedure")) AND (db:("LILACS" OR "BBO"))
LIVIVO https://www.livivo.de/	**#1** ("Lasers" OR "Laser Therapy" OR "Low Level Laser Therapy" OR "Low Level Light Therapy" OR "Photobiomodulation" OR "Photobiomodulation Therapy" OR "Laser Biostimulation" OR "Laser Phototherapy")
	**#2** ("Pain" OR "Pain Management" OR "Pain Perception" OR "Pain Measurement")
	**#3** ("Anesthesia" OR "Anesthesia, Dental" OR "Surgery, Oral" OR "Oral Surgical Procedures" OR "Oral Surgery" OR "Oral Procedure" OR "Dental Surgery" OR "Dental Procedures")
	**#1** AND **#2** AND **#3**
MEDLINE (*via* PubMed) http://www.ncbi.nlm.nih.gov/pubmed	#1 “Lasers”(Mesh) OR “Laser Therapy”(Mesh) OR “Low Level Laser Therapy”(tw) OR “Low Level Light Therapy”(tw) OR "Photobiomodulation"(tw) OR "Photobiomodulation Therapy"(tw) OR "Laser Biostimulation"(tw) OR "Laser Phototherapy"(tw)
	#2 "Pain"(Mesh) OR "Pain Management"(Mesh) OR "Pain Perception"(Mesh) OR "Pain Measurement"(Mesh)
	#3 "Anesthesia"(Mesh) OR "Anesthesia, Dental"(Mesh) OR “Oral Anesthesia”(tw) OR “Teeth Anesthesia”(tw) OR "Surgery, Oral"(Mesh) OR "Oral Surgical Procedures"(Mesh) OR "Oral Surgery"(tw) OR "Oral Procedure"(tw) OR "Dental Surgery"(tw) OR "Dental Procedures"(tw)
	#1 AND #2 AND #3
SciELO https://scielo.org/	(("Lasers" OR "Laser Therapy" OR "Low Level Laser Therapy" OR "Low Level Light Therapy" OR "Photobiomodulation" OR "Photobiomodulation Therapy" OR "Laser Biostimulation" OR "Laser Phototherapy") AND ("Pain" OR "Pain Management" OR "Pain Perception" OR "Pain Measurement") AND ("Anesthesia" OR "Anesthesia, Dental" OR "Surgery, Oral" OR "Oral Surgical Procedures" OR "Oral Surgery" OR "Oral Procedure"))
Scopus http://www.scopus.com/	(TITLE-ABS-KEY (("Lasers" OR "Laser Therapy" OR "Low Level Laser Therapy" OR "Low Level Light Therapy" OR "Photobiomodulation" OR "Photobiomodulation Therapy" OR "Laser Biostimulation" OR "Laser Phototherapy")) AND TITLE-ABS-KEY (("Pain" OR "Pain Management" OR "Pain Perception" OR "Pain Measurement")) AND TITLE-ABS-KEY (("Anesthesia" OR "Anesthesia, Dental" OR "Surgery, Oral" OR "Oral Surgical Procedures" OR "Oral Surgery" OR "Oral Procedure" OR "Dental Surgery" OR "Dental Procedures")))
Web of Science http://apps.webofknowledge.com/	**#1** TS=("Lasers" OR "Laser Therapy" OR "Low Level Laser Therapy" OR "Low Level Light Therapy" OR "Photobiomodulation" OR "Photobiomodulation Therapy" OR "Laser Biostimulation" OR "Laser Phototherapy")
	#2 TS=("Pain" OR "Pain Management" OR "Pain Perception" OR "Pain Measurement")
	#3 TS=("Anesthesia, Dental" OR "Oral Anesthesia" OR "Teeth Anesthesia" OR "Surgery, Oral" OR "Oral Surgical Procedures" OR "Oral Surgery" OR "Oral Procedure" OR "Dental Surgery" OR "Dental Procedures")
	#1 AND #2 AND #3
Gray Literature	
EASY https://easy.dans.knaw.nl/	("Lasers" OR "Dental Anesthesia")
Google Scholar https://scholar.google.com.br/	("Lasers") AND ("Dental Anesthesia") filetype:pdf
OATD http://www.oatd.org/	(("Lasers") AND ("Anesthesia"))

The obtained results were exported to the EndNote Web™ software (Clarivate™ Analytics, Philadelphia, USA), in which duplicates were removed automatically, and the remaining ones were removed manually. The other results were exported to Rayyan QCRI (Qatar Computing Research Institute, Doha, Qatar) [20] for the study selection phase. The manual analysis of the gray literature occurred simultaneously and fully using Microsoft Word™ 2010 (Microsoft™ Ltd., Washington, USA).

Before selecting the studies, two reviewers performed a calibration exercise in which they discussed the eligibility criteria and applied them to a sample of 20% of the retrieved studies to determine inter-examiner agreement. The selection started after reaching an adequate level of agreement (Kappa ≥0.81) and occurred in two phases.

In the first phase, two eligibility reviewers (CMM and MBO) methodically analyzed the titles and abstracts of the studies independently. A third examiner (MDMAC) investigated and solved disagreements between the reviewers. Titles unrelated to the topic were eliminated in this phase as well as abstracts, respecting the eligibility criteria. In the second phase, the full texts of the preliminarily eligible studies were obtained and evaluated. If the full texts were not found, a bibliographic request was made to the library database (COMUT), and an e-mail was sent to the corresponding authors to obtain the texts.

### Data collection

A calibration exercise was performed before data extraction to ensure consistency between the reviewers, in which the data from three eligible studies were extracted jointly. After the calibration, two reviewers (CMM and MBO) extracted the data from the eligible studies, independently and blinded. A third reviewer (MDMAC) analyzed the conflicts in cases of disagreement about data extraction.

The following data were extracted from the articles: study identification (author, year, country, location, and application of ethical criteria), sample characteristics (sample size, distribution by sex and average age, laser specifications, laser application protocol, and anesthetic technique protocol), collection and processing characteristics (pain assessment tool, time of pain assessment, and type of statistical analysis), and main results (overall pain score from each pain assessment tool applied to each group and main results of each study). In case of incomplete or insufficient data, the corresponding authors were contacted via e-mail up to three times at weekly intervals.

### Risk of bias assessment

Two reviewers (WAV and CMM) independently assessed the risk of bias in the selected studies using the Cochrane Collaboration Risk of Bias tool (version 2.0) (RoB2) for RCTs [21]. This instrument consists of five domains: bias from the randomization process, bias due to deviations from intended interventions, bias from missing outcome data, bias in outcome measurement, and bias in the selection of reported results.

The evaluation of each domain followed the algorithms proposed by the RoB2 manual [21]. Any disagreements between the reviewers were resolved by discussing and consulting with a third reviewer (LRP).

### Summary measures and synthesis of results

The data collected from the selected studies were organized in spreadsheets on Microsoft Excel™ 2019 (Microsoft™ Ltd., Washington, USA) and described narratively (qualitative synthesis). The quantitative results of pain assessment tools applied to patients after local anesthetic puncture were described for measuring pain perception. A meta-analysis was planned but not performed due to the high heterogeneity of the studies.

### Certainty of evidence (GRADE approach)

Two reviewers (WAV and MBO) independently ranked the overall strength of evidence using the Grading of Recommendations, Assessment, Development, and Evaluation (GRADE) tool [22]. To assess the criteria in systematic reviews without meta-analyses, the authors followed the adaptations by Murad et al. [23].

## Results

### Study selection

The electronic search identified 3,485 results distributed into eight electronic databases, including the “gray literature”. After removing duplicates, 2,268 results remained for the analysis. A careful reading of the titles and abstracts excluded 2,214 results.

**Fig. 1:**
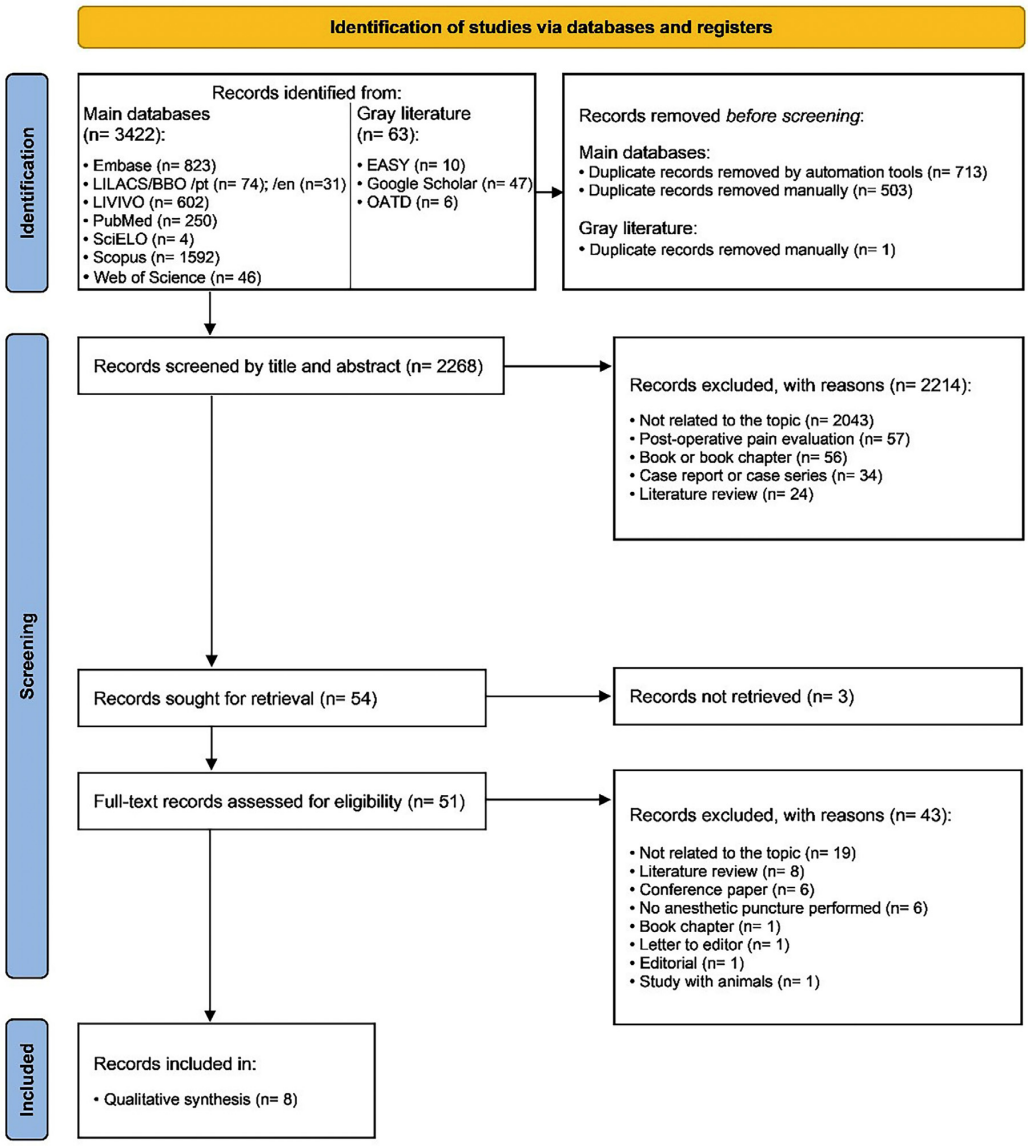
Flowchart of the literature search and selection, adapted from the preferred reporting items for systematic reviews.

After reading the full texts, 46 studies were excluded, and eight [24], [25], [26], [27], [28], [29], [30], [31] were included in the qualitative synthesis. Fig. 1 details the study selection process.

### Study characteristics

The articles were published from 2011 to 2022 and performed in six countries: five studies in Asia [25,26,[28], [29], [30]], one in Europe [27], one in South America [24], and one in a transcontinental Asia-Europe country – Turkey [31]. The sum of eligible study participants resulted in 540 patients. The age groups in the eligible studies ranged from six [31] to 75-years [27], and male patients composed most samples in studies that sampled by sex [[25], [26], [27], [28], [29],^31^].

All studies used diode lasers with wavelengths from 660 nm to 980 nm [24], [25], [26], [27], [28], [29], [30], [31]. Regarding laser application protocols, the puncture site surface was prepared by isolating and drying the mucosa in one study [30] and applying topical anesthetic gel in two other studies [26,31] before laser application. The other studies did not specify surface preparations before laser application [24,25,[27], [28], [29]]. The laser application time was from 20 s [29,31] to three minutes [28]. Light emission was continuous in five studies [[25], [26], [27],^29^,^31^] and pulsed in one [30], and two studies did not report this specification [24,28]. Only three studies specified the laser-surface distance: in contact [25], 1 mm away [31], and 2 mm away [30].

As for anesthetic techniques, five studies performed the anesthetic puncture in the maxilla [[24], [25], [26], [27],^30^], one in the mandible [31], and one in both [27], and one study did not specify the injection site [28]. Only one study did not administer a local anesthetic when performing the injection [26].

Regarding pain assessment tools, one study used the Visual Numerical Scale (VNS) [24], four used the Visual Analog Scale (VAS) [25,26,28,29], one used the Numerical Rating Scale (NRS) [27], two used the Wong-Baker Faces Pain Rating Scale (WBFPRS) [30,31], one used the Sound, Eye, and Motor (SEM) scale [30], and one study used the Face, Legs, Activity, Cry, and Consolability (FLACC) scale [31].

Table 2 presents the main characteristics of each eligible article.

**Table 2:** Main characteristics of eligible studies.

Author, year (country)	Study design	Sample (♀, ♂)	Mean age ± SD	Laser specifications (brand of device)	Laser application protocol	Anesthetic technique protocol	Pain assessment method
Dantas et al., 2011 (Brazil) [24]	Parallel	60 (nr) 20 - Control 1: light curing 20 - Control 2: no treatment 20 - Test: PBMT	nr	Gallium aluminum arsenide	Applied at the anesthetic puncture site for 33 s.	Injection of ^1^/_4_ of the local anesthetic cartridge (Novocol, SS White) in the palate region, approximately 3 mm above the cervical region of the teeth to be anesthetized.	10-score VNS applied after injection.
				Wavelength: 830 nm			
				Power: 120 mW			
				Focal spot: 2 mm^2^			
				Energy density: 4 J/cm^2^			
Sattayut, 2014 (Thailand) [25]	Parallel	80 (40♀, 40♂) 20 - Control: topical anesthesia 20 - Test: Pressure 20 - Test: PBMT without radiation 20 - Test: PBMT	21 ± nr a18 to 25 years old	Wavelength: 790 nm	Applied at the anesthetic puncture site for 2 min in contact mode.	Injection of 0.5 mL of 2% lidocaine hydrochloride solution with 1:100,000 epinephrine into the anesthetized area using a pressure and volume control intraligamental syringe and a 27-gauge disposable needle (Citojet, Bayer, Germany).	100 mm VAS applied after injection.
				Continuous mode			
				Power: 30 mW			
				Focal spot: 0.13 cm^2^			
				Energy density: 27.69 J/cm^2^			
Ghaderi et al., 2016 (Iran) [26]	Split-mouth	66 (30♀, 36♂) 66 - Control: topical anesthesia 66 - Test: topical anesthesia + PBMT	23.34 ± 2.16	Gallium Aluminum Arsenide (AZERO K2, Russia)	Mucosa was isolated and dried for 30 s with a cotton. Local anesthetic gel (benzocaine) was applied for 60 s. Photobiomodulation was applied at the anesthetic puncture site for 1 min.	Insertion of a 27-gauge short needle into the buccal mucosa of the maxillary canine region after laser irradiation for the laser group and after application of topical anesthetic for the placebo group.	100 mm VAS applied after needle insertion.
				Wavelength: 960 nm			
				Continuous mode			
				Power: 100 mW			
				Focal spot: nr			
				Energy density: 4 J/cm²			
Tuk et al., 2017 (Netherlands) [27]	Parallel	163 (82♀, 81♂) 80 – Control: no treatment 83 – Test: PBMT	L (32 ± 14); P (29 ± 12); a18 to 75 years old	LX2 Control Unit with laser probe (THOR Photomedicine Ltd., Chesham, UK)	Both maxilla and mandible had 2 target-sites irradiated for 30 s continuously, and each target-site was also irradiated twice in a row. Total irradiation time was 2 min (1 min for each target-site) with a 30 s interval between each irradiation.	Local anesthesia or mandibular block was performed with 4% articaine hydrochloride with 1:100,000 epinephrine (1.7-mL syringe Ultracain D-S forte, Sanofi-Aventis Netherlands BV, Gouda, Netherlands), using a 27-G needle (Terumo 27 13/8, Somerset, NJ).	11-point NRS applied before and immediately after injection.
				Wavelength: 810 nm			
				Continuous mode			
				Power: 198 mW			
				Focal spot: 0.088 cm^2^			
				Energy density: 67.5 J/cm^2^			
Jagtap et al., 2019 (India) [28]	Split-mouth	25 (12♀, 13♂) 25 – Control: PBMT without radiation 25 – Test: PBMT	28.3 ± 5.5 a18 to 60 years old	Microcontroller Based Diode Laser (Silberbauer, India)	Applied at the anesthetic puncture site for 3 min.	Standard local anesthetic was injected to perform the blocking technique.	10-score VAS applied after injection.
				Wavelength: 660 nm			
				Power: 60 mW			
				Focal spot: nr			
				Energy density: nr			
Ghabraei et al., 2020 (Iran) [29]	Parallel	56 (29♀, 27♂) 22 – Control: PBMT without radiation 12 – Control: no treatment 22 – Test: PBMT	L (39.2 ± 12.2) P (37.6 ± 12) C (37.8 ± 11) a18 to 60 years old	Simpler Diode Laser (Doctorsmile, Italy)	Applied at the anesthetic puncture site (buccal mucosa) for 20 s continuously.	A tube of 2% lidocaine with 1:80,000 epinephrine (Daroo Pakhsh, Iran) was injected in the anterior buccal region of the maxilla completely at a rate of 1 mL/min with a 27-gauge short needle (Technofar, Italy).	170 mm VAS.
				Wavelength: 980 nm			
				Power: 300 mW			
				Focal spot: 0.384 cm²			
				Energy density: 15.62 J/cm²			
Amruthavarshini et al., 2021 (India) [30]	3-arm, crossover	30 (NR) 30 - Control: topical anesthesia 30 - Test 1: Ice application 30 - Test 2: PBMT	Nr a9 to 12 years old	DenLase Diode Laser (China Daheng Group, Inc., China)	Mucosa was properly isolated and dried. Photobiomodulation was applied at anesthetic puncture site for 1 min in pulsed mode and 2 mm away from the surface.	An injection of local anesthetic solution (Lignox 2% A, Kilitch Drugs India Ltd., Navi Mumbai) was performed at 1 mL/min.	WBFPRS and SEM scales applied during injection.
				Wavelength: 810 nm			
				Pulsed mode			
				Power: 300 mW			
				Focal spot: nr			
				Energy density: nr			
Uçar et al., 2022 (Turkey) [31]	Split-mouth	60 (30♀, 30♂) 60 - Control: topical anesthesia 60 - Test: topical anesthesia + PBMT	7.11 ± 1.12 a6 to 9 years old	Cheese Dental Diode Laser (GIGAA LASER, Wuhan Gigaa Optronics Technology Co., China)	Mucosa was properly isolated and dried. Local anesthetic gel (10% lidocaine) was applied for 60 s with cotton. Photobiomodulation was applied at anesthetic puncture for 20 s with slow circular movements and 1-mm away from the surface.	Local anesthesia was performed with 1-mL of anesthetic solution containing 4% articaine hydrochloride with 1:100,000 epinephrine (Ultracaine D-S forte, Hoechst Canada Inc., Montreal Quebec, Canada), a 27-gauge dental needle and a 2-mL plastic disposable dental syringe (Helmed, Adana, Turkey).	WBFPRS and FLACC scales applied immediately after injection in two stages: 1) During needle insertion; 2) During anesthetic solution deposition.
				Wavelength: 810 nm			
				Continuous mode			
				Power: 300 mW			
				Focal spot: 0.087 cm^2^			
				Energy density: 69 J/cm^2^			

nr, Not Reported in the study; PBMT, Photobiomodulation Therapy; VNS, Visual Number Scale; VAS, Visual Analog Scale; NRS, Numerical Rating Scale; WBFPRS, Wong-Baker Faces Pain Rating Scale; SEM, Sound, Eyes, and Motor; FLACC, Face, Legs, Activity, Cry, and Consolability.

a Age range.

### Individual results of the studies

Five studies compared the application of PBMT versus placebo in adults [24,25,[27], [28], [29]]. Among these, four articles did not find significant differences between the groups, and one [28] concluded that the PBMT group had lower pain scores during the anesthetic puncture. Two studies [26,31] evaluated PBMT associated with a topical anesthetic versus topical anesthetic application alone - one found no difference between groups and the other observed better results for the PBMT group only using the WBFPRS. Two studies [26,30] also compared PBMT versus topical anesthetic, and PBMT presented similar or worse results than topical anesthetic. Table 3 shows details of the outcomes and conclusions of each eligible study.

**Table 3:** Main results of eligible studies.

Author	Pain assessment tool	Groups (sample size)	Overall pain score ± SD	Main conclusion
Dantas et al. [24]	10-score VNS	G1 – Laser group (n=20)	1.9 ± nr	PBMT reduced the resulting pain of anesthetic application in the palate region when used before the application.
		G2 – Placebo group (n=20)	2.7 ± nr	
		G3 – Control group (n=20)	4.35 ± nr	
Sattayut [25]	100 mm VAS	G1 – Laser group (n=20)	11 (10‒30)^c^	There were no statistically significant differences in pain scores among groups.
		G2 – Local anesthetic gel group (n=20)	23 (18‒39)^c^	
		G3 – Pressure group (n=20)	27 (12‒35)^c^	
		G4 – Light touch group (n=20)	31 (13‒38)^c^	
Ghaderi et al. [26]	100 mm VAS	G1 – Laser group (n=66)^a^	21 ± 2.9	PBMT was not effective in decreasing pain perception due to needle insertion into the maxillary buccal mucosa.
		G2 – Placebo group (n=66)^a^	19 ± 2.7	
Tuk et al. [27]	11-point NRS	G1 – Laser group (n=83)	5.2 ± 2.4	PBMT did not effectively decrease pain during local anesthetic injections before third molar surgery.
		G2 – Placebo group (n=80)	4.8 ± 2.2	
Jagtap et al. [28]	10-score VAS	G1 – Laser group (n=25)^a^	2.8 ± 0.866	PBMT reduced pain during injection of local anesthesia.
		G2 – Placebo group (n=25)^a^	7.12 ± 1.301	
Ghabraei et al. [29]	170 mm VAS	G1 – Laser group (n=22)	88.1 ± 34.5	PBMT significantly reduced the local anesthesia injection pain in the anterior maxillary region without superiority over placebo irradiation.
		G2 – Placebo group (n=22)	95 ± 33.4	
		G3 – Control group (n=12)	101.1 ± 22	
Amruthavarshini et al. [30]	WBFPRS	G1 – Laser group (n=30)^b^	2.13 ± 1.66	PBMT was less effective than the other two techniques.
		G2 – Ice group (n=30)^b^	1.13 ± 1.36	
		G3 – Local anesthetic gel group (n=30)^b^	1.27 ± 1.34	
	SEM scale	G1 – Laser group (n=30)^b^	1.67 ± 0.61	
		G2 – Ice group (n=30)^b^	1.07 ± 0.25	
		G3 – Local anesthetic gel group (n=30)^b^	1.23 ± 0.43	
Uçar et al. [31]	WBFPRS	G1 – Needle insertion in laser group (n=60)^b^	0.82 ± 0.83	PBMT showed significantly better results only using the Wong-Baker Faces Pain Rating Scale.
		G2 – Needle insertion in control group (n=60)^b^	1.23 ± 0.92	
		G3 – Anesthetic deposition in laser group (n=60)^b^	0.82 ± 0.83	
		G4 – Anesthetic deposition in control group (n=60)^b^	1.23 ± 0.92	
	FLACC scale	G1 – Needle insertion in laser group (n=60)^b^	0.67 ± 0.6	
		G2 – Needle insertion in control group (n=60)^b^	0.8 ± 0.66	
		G3 – Anesthetic deposition in laser group (n=60)^b^	0.67 ± 0.6	
		G4 – Anesthetic deposition in control group (n=60)^b^	0.78 ± 0.67	

nr, Not Reported in the study; PBMT, Photobiomodulation Therapy; VNS, Visual Number Scale; VAS, Visual Analog Scale; NRS, Numerical Rating Scale; WBFPRS, Wong-Baker Faces Pain Rating Scale; SEM, Sound, Eyes, and Motor; FLACC, Face, Legs, Activity, Cry, and Consolability

a Split-mouth sample.

b Crossover sample.

c Median; (number – number) ‒ confidence interval.

### Risk of individual bias in the studies

**Fig. 2:**
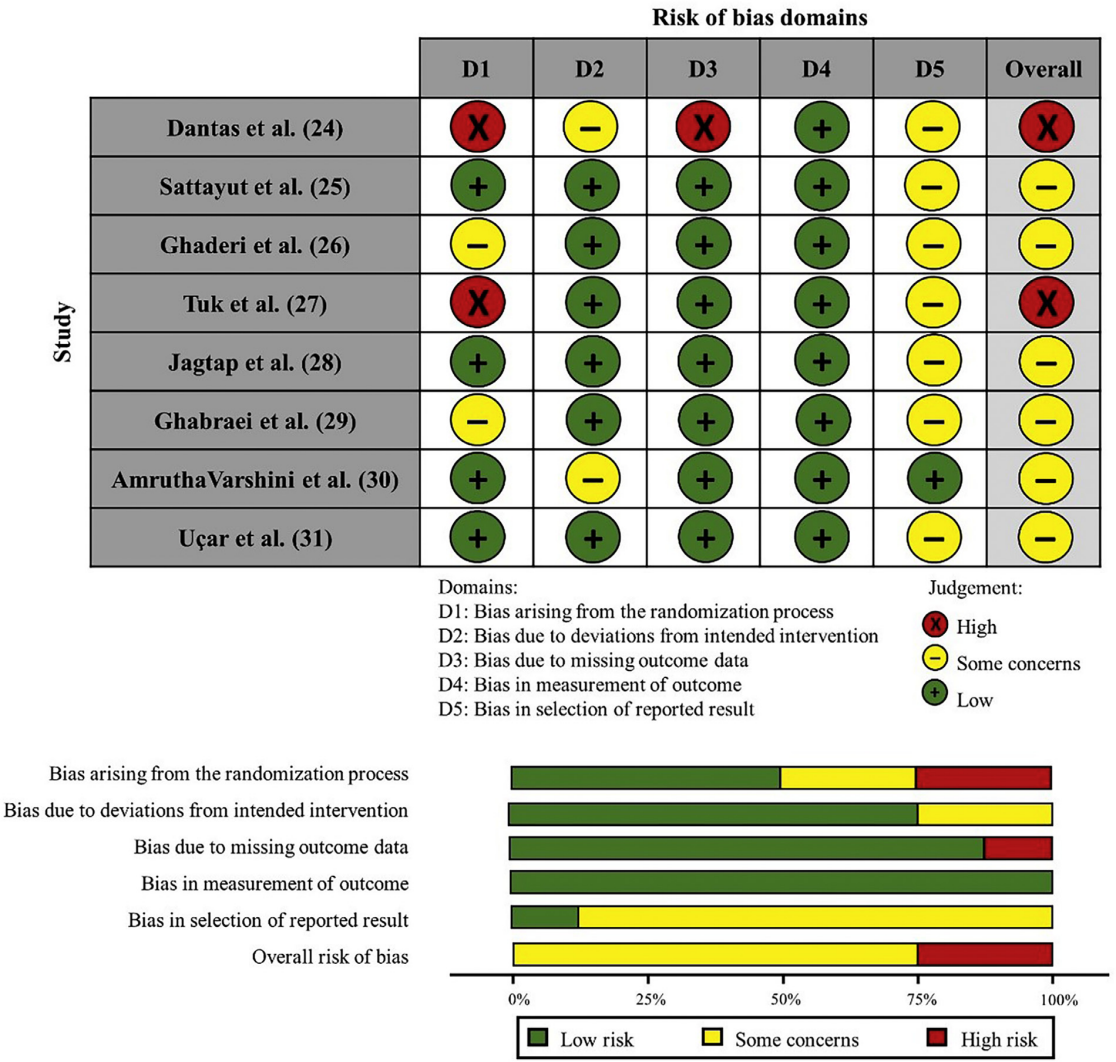
Individual risk of bias assessment.

Among the eight studies, two [24,27] were classified as a “high risk of bias” and six [25,26,[28], [29], [30], [31]] as “some concerns”. Most studies presented biases regarding the randomization process [24,26,27,29] and selection of reported results [[24], [25], [26], [27], [28], [29],^31^], and only one [24] showed a high risk of bias in the “missing outcomes” domain. Fig. 2 shows the individual assessment of each included article.

### Certainty of evidence

The certainty of evidence analysis considered the interventions and age of the population. The outcomes presented very low to low certainty of evidence. Table 4 shows details of the individual assessment of each outcome.

**Table 4:** Summary of findings by the Grading of Recommendations Assessment, Development, and Evaluation (GRADE) for the systematic review outcomes.

**GRADE assessment**		
**Number of studies (participants)**	**Summary of findings**	**Certainty**
***PBMT versus Placebo (Adults)***		
5 (344 participants)	Four studies did not find any statistical difference between placebo and PBMT, one study found better results for the PBMT group.	⨁⨁ Low^a^^,^^b^
***PBMT plus topic anesthetic versus Topic anesthetic (Adults)***		
1 (66 participants)	The study did not find significant differences between groups.	⨁ Very low^a^^,^^c^^,^^d^
***PBMT plus topic anesthetic versus Topic anesthetic (Children)***		
1 (60 participants)	The study showed significantly better results for the PBMT group only using the Wong-Baker Faces Pain Rating Scale.	⨁⨁ Low^c^^,^^d^
***PBMT versus Topic anesthetic (Adults)***		
1 (80 participants)	The study did not find significant differences between the groups.	⨁⨁ Low^c^^,^^d^
***PBMT versus Topic anesthetic (Children)***		
1 (30 participants)	PBMT group presented significantly higher pain scores.	⨁ Low^c^^,^^d^

PBMT, Photobiomodulation Therapy.

a Risk of bias ‒ One or more studies presented a high risk of bias.

b Imprecision ‒ Few participants (<400) – downgraded by one level.

c Imprecision – Very few participants (<100) ‒ downgraded by two levels.

d Inconsistency was not assessed because there was only one study included. GRADE Working Group grades of evidence High certainty: Very confident that the true effect is close to the estimated effect. Moderate certainty: Moderately confident in the estimated effect: The true effect is likely close to the estimated effect, but it may be substantially different. Low certainty: Limited confidence in the estimated effect: The true effect may be substantially different from the estimated effect. Very low certainty: Very little confidence in the estimated effect: The true effect may be substantially different from the estimated effect.

## Discussion

Dental local anesthesia can reduce pain from procedures performed in patient's mouths, but local anesthetic puncture is often related to the experience of pain and anxiety. Pain perception during the anesthetic technique is usually noticed in two moments: at needle insertion and local anesthetic deposition [31]. There are numerous resources to make local anesthesia more comfortable, such as local anesthetic gels [32], computerized local anesthesia systems [33], behavioral management techniques [34], acupuncture combined with conventional treatments [35], anesthetic puncture site pre-cooling [36], and some clinical trials have already been conducted to test PBMT as one of these resources. Therefore, this study aimed to analyze the effect of photobiomodulation therapy on pain perception during the anesthetic puncture of dental local anesthesia.

The studies in this review included participants from different age groups: children [31], children and adolescents [30], and adults [24], [25], [26], [27], [28], [29]. Studies with children and adolescents showed controversial pain scores [30,31]. That may be supported because of the challenge in assessing pain in children due to their very subjective verbal and non-verbal communication, which relates to sensory, emotional, cognitive, and social factors [37]. Additionally, younger individuals may often confuse felt stimuli, such as needle pressure or touch, with pain perception. Adults had more homogeneous results in the laser groups with slightly lower pain levels [24,25,28,29], except for two studies [26,27]. That emphasizes the need for caution when linking subjective experiences to only a specific age group, considering that older groups may also have individual perspectives of pain and anxiety. Another discussion topic is the difference between pain perception regarding the procedure site and puncture and the anesthetic, infiltrative, or blocking techniques. Palatal anesthesia is more painful than in other regions in the mouth because the palatal tissues are relatively noncompliant and tightly bound to the periosteum [38,39], potentially benefiting more from PBMT. However, there were conflicting results between the two studies evaluating palatal anesthesia [24,25]. Anesthetic techniques with higher needle penetration and bone touch as a reference for needle penetration depth, such as block anesthesia, would tend to cause more pain, which the studies did not confirm [27,28].

The studies also used different laser application protocols. The wavelengths varied among the studies but were all within the visible red and near-infrared spectrum (390‒1600 nm), agreeing with the therapeutic window for the clinical benefits of PBMT described in the literature [8]. There is no agreement on surface preparation, as only two studies reported drying and applying a topical anesthetic gel before laser application [26,31]. Furthermore, the results of the association of topical anesthetic gels with PBMT were controversial between these two studies [26,31]. Therefore, no standardized protocol can be defined, but removing surface residues may be the minimum operators must do to maintain a clean operative field before laser application. Research studies may also recommend against using local anesthetic gels before laser application because it can represent a confounding factor in analgesic effect evaluations. Topical anesthesia is a proven adjunct to pain control in local anesthetic delivery [40], potentially masking the actual effect of PBMT at the puncture site. However, the controversial results regarding the previous application of topical anesthesia may be due to heating and increased local metabolism from PBMT, which could cause a loss of effectiveness of the local anesthetic.

The results of eligible studies showed an interesting analgesic effect curve related to the time of photobiomodulation application. Application times of around 30 s [24,29,31] showed lower pain perception than placebo and control groups but without significant differences. After one minute of application [26,27,30]. The comparators overcame this effect. Interestingly, the laser groups returned to show better results than other study groups after two to three minutes of continuous application [25,28]. That can be partially explained by the Arndt-Schulz law, which recognizes a biostimulation threshold that, when reached, can inhibit the effect [8]. However, the return of the positive analgesic outcome after a longer application time remains a compelling finding that future studies should explore.

Although there is no consensus on the light-emitting mode for PBMT, the literature highlights the pulsed one, as it generates less heat to tissues [8]. However, eligible studies did not provide sufficient evidence to assert the superiority of any light-emitting mode for analgesic effect. The reports of laser-surface contact in three studies [25,30,31] allow inferring us that better analgesic results of PBMT may be associated with a closer contact distance between the PBMT device tip and the irradiated surface. Further studies are required to provide more evidence regarding the light-emitting and laser-surface contact modes.

Regarding the location of PBMT application, the irradiation sites slightly differed between the studies, and all were intraoral and close to the anesthetic puncture site. Choosing these sites of application is logically consistent with the theory of photobiomodulation therapy mechanism via tissue biomodulation by triggering molecular processes, and generating local analgesia in other clinical situations [8]. Interestingly, the literature describes the irradiation of the LI4 acupuncture point on the hand, showing an analgesic outcome during dental local anesthetic puncture [41]. Acupuncture can control procedure pain in pediatric patients, and practitioners have been increasingly interested in alternative techniques. Acupuncture combined with conventional methods can help achieve better patient experience and procedure outcomes [42].

There are insufficient current data to confirm an association between local anesthetic techniques and PBMT regarding the pain perception from dental local anesthetic puncture. The studies applied procedures in the maxilla and the mandible and did not show solid evidence of the superiority of the analgesic effect of PBMT for a specific technique. That might be due to an actual absence of effect superiority or insufficient study homogeneity to establish a comparison. For instance, local anesthetic gels have proven superior in relieving pain during anesthetic techniques performed in the maxilla compared to those in the mandible [32]. Therefore, further standardized studies are needed comparing different anesthetic techniques after photobiomodulation.

Pain assessment tools from eligible studies highly differed. There were VAS variations: 100 mm VAS [25,26], 10-score VAS [28], and 170 mm VAS [29]. Studies with the same scales, such as 100 mm VAS [25,29] and WBFPRS [30,31], had other relevant methodological differences, such as using topical anesthetic gels [26,31]. Even so, the literature describes all scales used in eligible studies as reliable pain assessment tools objectively (VNS, VAS, NRS, and WBFPRS) and subjectively (FLACC and SEM scales) [2], [3], [4], [5], [6], [7].

Regarding the risk of individual bias in the eligible studies, the most frequent were methodological biases of the randomization and selection of reported results. Randomization should be present to reduce the risk of known participant characteristics affecting study group allocation, as it can distort comparability between groups. Thus, the lack of a clear description of the used randomization technique configures a bias due to the uncertainty that the study followed this principle. Additionally, two studies [24,30] raised concerns about participant blinding during the trial. As pain is a subjective experience, blinding participants is crucial to avoid overestimating the results due to the awareness of an intervention.

This study has limitations that must be acknowledged. The included studies presented characteristics that limited the performance of a meta-analysis, such as different pain assessment tools, laser application protocols, and age groups. There was at least one heterogeneity factor between the two studies. Hence, a meta-analysis could not be performed. Furthermore, the lack of a homogeneous methodology and the presence of a confounding bias hindered the consensus of a completely standardized protocol for the analgesic effect of PBMT. That highlights the need for further randomized controlled trials with standardization of methodological execution and reporting protocols to allow a deeper interpretation of findings.

## Conclusion

Based on a low to very low certainty of evidence, PBMT seems to have no effect on pain perception during the anesthetic puncture in patients undergoing dental local anesthesia. Therefore, further randomized studies with a low risk of bias should be performed with a standardized methodology regarding the execution and reporting of photobiomodulation therapy specifications.

## Ethics

Not applicable.

## Funding

This study was partially funded by the Coordination for the Improvement of Higher Education Personnel ‒ Brazil (CAPES) – Finance Code 001. The authors also appreciate the support from the Counsel of Technological and Scientific Development ‒ Brazil (CNPq) and FAPEMIG.

## CRediT authorship contribution statement

**Caio Melo Mesquita:** Conceptualization, Investigation, Methodology, Data curation, Validation, Writing – original draft, Writing – review & editing. **Millena Barroso Oliveira:** Conceptualization, Investigation, Methodology, Data curation, Validation, Writing – original draft, Writing – review & editing. **Marcelo Dias Moreira de Assis Costa:** Conceptualization, Investigation, Methodology, Data curation, Validation, Writing – original draft, Writing – review & editing. **Walbert Andrade Vieira:** Methodology, Data curation, Validation, Writing – original draft, Writing – review & editing, Supervision. **Rafael Rodrigues Lima:** Methodology, Data curation, Validation, Writing – review & editing, Supervision. **Sigmar de Mello Rode:** Methodology, Data curation, Validation, Writing – review & editing, Supervision. **Luiz Renato Paranhos:** Conceptualization, Investigation, Methodology, Data curation, Validation, Writing – original draft, Writing – review & editing, Supervision.

## Conflicts of interest

All authors certify that they have no affiliations with or involvement in any organization or entity with any financial or non-financial interest in the subject matter or materials discussed in this manuscript.
